# Diagnosis and Management of Graves' Disease in Thailand: A Survey of Current Practice

**DOI:** 10.1155/2020/8175712

**Published:** 2020-05-11

**Authors:** Chutintorn Sriphrapradang

**Affiliations:** Department of Medicine, Faculty of Medicine Ramathibodi Hospital, Mahidol University, Bangkok 10400, Thailand

## Abstract

**Background:**

The data on clinical practice patterns in the evaluation and management of Graves' disease (GD) are limited in Asia. The aims of this survey were to report the current practices in the management of GD in Thailand and to examine any international differences in the management of GD.

**Methods:**

Members of the Endocrine Society of Thailand who were board certified in endocrinology (*N* = 392) were invited to participate in an electronic survey on the management of GD using the same index case and questionnaire as in previous North American and European surveys.

**Results:**

One hundred and twenty responses (30.6%) from members were included. TSH receptor antibody measurement (29.2%), thyroid ultrasound (6.7%), and isotopic studies (5.9%) were used less frequently to confirm the etiology compared with those in North American and European surveys. Treatment with an antithyroid drug (ATD) was the preferred first choice of therapy (90.8%). Methimazole at 10–15 mg/day with a beta-blocker was the initial treatment of choice. The preferred ATD in pregnancy was propylthiouracil in the first trimester and methimazole in the second and third trimesters, which was similar to the North American and European surveys.

**Conclusion:**

Ultrasound and isotopic studies will be requested only by a small proportion of Thai endocrinologists. Higher physician preference for ATD is similar to Europe, Latin America, and other Asian countries. Geographical differences in the use of ATD, radioactive iodine, and thyroidectomy exist.

## 1. Background

Graves' disease (GD) is the most common cause of hyperthyroidism in iodine-replete areas [1]. The development of GD is thought to be due to complex interactions between genetic and environmental factors. Its autoimmune origin is well known, and the stimulation of autoantibodies to the TSH receptor (TRAb) on thyroid follicular cells is responsible for hyperthyroidism and development of a goiter. The clinical features of GD are shared by other etiologies of thyrotoxicosis. However, GD is associated with distinct extrathyroidal manifestations, including Graves' orbitopathy (GO), thyroid dermopathy, and acropachy. The diagnosis of GD can often be established on the basis of the clinical presentation, raised levels of thyroxine (T4), and suppressed levels of TSH. If the diagnosis is not straightforward, supplementary testing may include TRAb measurement, a radioactive iodine (RAI) uptake test, or color-flow Doppler ultrasonography of the thyroid gland [2, 3]. The three therapeutic approaches for treating patients with GD are antithyroid drugs (ATDs), a RAI therapy, and surgical thyroidectomy. All three treatment options are effective, but each treatment approach has advantages and drawbacks. Patient-centered communication and shared decision making are becoming increasingly important in determining the most suitable treatment option. The treating physician and patients should discuss the logistics, cost of care, expected recovery time, benefits, disadvantages, and possible side effects for each of the treatment options. The decision may also be influenced by the severity of thyrotoxicosis.

Persistent marked variations in the diagnosis and management of GD exist throughout the world [4]. Burch and colleagues conducted a 2011 questionnaire-based survey of actual clinical practice in the management of GD among international members of the Endocrine Society, the American Association of Clinical Endocrinologists, and the American Thyroid Association (ATA) [5]. In addition, a similar survey was performed in 2013 among members of the European Thyroid Association (ETA) [6]. In Asia, the results of surveys on clinical practice patterns in the management of GD are available only from Japan, Korea, China, and India [7, 8]. To this purpose, we used the same questionnaire developed by Burch et al. [5] and distributed it among members of the Endocrine Society of Thailand (EST) to investigate the clinical practice patterns in the management of GD in Thailand.

## 2. Methods

### 2.1. Survey

A survey administration application (Google Forms, Mountain View, CA, USA) was used to administer the survey. The survey included the index case (a 42-year-old woman with uncomplicated GD) with two variants, including a patient with GO and a patient anticipating pregnancy in the next 6–12 months, and the same questions as in the earlier surveys [5]. Description of the index case was “a 42-year-old woman presents with moderate hyperthyroid symptoms of 2 months duration. She is otherwise healthy, takes no medications, and does not smoke cigarettes. She has two children, the youngest of whom is 10 years old, and does not plan on being pregnant again. This is her first episode of hyperthyroidism. She has a diffuse goiter, approximately two to three times normal size, pulse rate of 105 beats per minute, and has a normal eye examination. Thyroid hormone levels are found to be twice the upper limit of normal (free T4 3.6 ng/dL, normal range 1.01–1.79 ng/dL), with an undetectable thyrotropin level (TSH <0.01 mIU/L).” Most questions required a single best response to be selected from multiple choices. Diagnostic preference questions allowed multiple items to be simultaneously selected. To limit bias, questions were carefully constructed to exclude phrasing that could influence the respondents' answers. The study was approved by the Committee on Human Rights Related to Research Involving Human Subjects of the Faculty of Medicine Ramathibodi Hospital, Mahidol University. The study was conducted in accordance with the principles of the Declaration of Helsinki and Good Clinical Practice guidelines.

Members of the EST who are board certified in endocrinology (*N* = 392) received e-mails from the EST administrators that included an electronic link to the questionnaire. The authors did not contact potential respondents directly. Survey responses were anonymously collected and stored electronically by the survey application service, accessible in a password-protected manner. Repeat submissions from the same Internet protocol address were automatically blocked by the survey service. Responses were then compared with those of 457 North American specialists extracted from the 2011 American survey [5] and 147 ETA members extracted from the 2013 survey [6]. The responses from respondents were collected from 26 June 2019 to 17 August 2019.

### 2.2. Statistical Analysis

Summary statistics were prepared for responses to each question. Data were analysed using STATA software version 14 (StataCorp LP, TX, USA).

## 3. Results

### 3.1. Response Rate and Respondent Demographics

One hundred and twenty respondents (about 30.6% of the members of the EST who were board certified in endocrinology) participated in the survey, and 100% completed all sections. Due to lacking our own guidelines, respondents mostly followed ATA guidelines. Most respondents graduated from medical school in the 2000s (57%) with 22% graduating in the 2010s and 15% graduating by the 1990s. Forty-seven percent were currently working in a medical school, 28% were working in a private hospital, and 25% were working in a secondary or tertiary healthcare center. Eighty-eight percent of the respondents reported treatment of >10 new cases of GD yearly.

### 3.2. Diagnostic Evaluation of the Index Case

**Figure 1:**
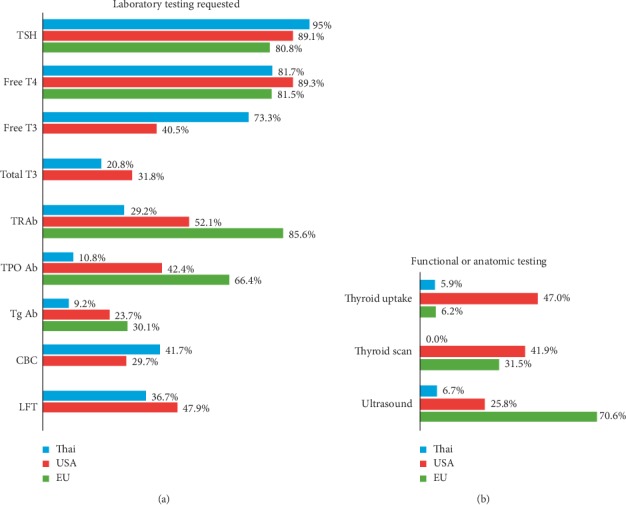
Percentage of participants who would obtain the listed laboratory test (a) or functional and anatomic study (b) in a patient with uncomplicated Graves' disease. International differences in the selection of laboratory tests or imaging studies are also shown. USA and EU data are from references 5 and 6, respectively. CBC, complete blood count; EU, Europe; LFT, liver function test; T3, triiodothyronine; T4, thyroxine; Tg Ab, thyroglobulin antibody; TPO Ab, thyroperoxidase antibody; TRAb, TSH receptor antibody; USA, United States of America.

Figure 1(a) shows the proportion of respondents requesting the listed laboratory investigations for the index case. Serum TSH and free T4 assays were the most frequently ordered measurements (95% and 81.7%, respectively), whereas serum free triiodothyronine (T3) or total T3 were less frequently requested (73.3% and 20.8%, respectively). In the initial evaluation of GD, serum TRAb measurements were requested by the minority of respondents (29.2%), whereas thyroperoxidase antibody (TPO Ab) and thyroglobulin antibody (Tg Ab) tests were ordered less frequently (10.8% and 9.2%, respectively).

Figure 1(b) shows the proportion of respondents who ordered the listed anatomical or functional investigations for the index case. Thyroid ultrasound and RAI uptake were requested by 6.7% and 5.9%, respectively. Baseline assessments of the complete blood count (CBC) and liver function tests were acquired by 41.7% and 36.7% of the respondents, respectively.

### 3.3. Therapy

#### 3.3.1. Preferred First-Line Treatment in the Index Case

**Figure 2:**
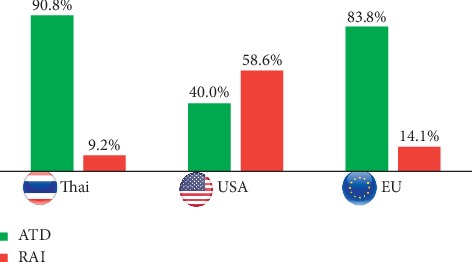
International differences in the selection of primary treatment modalities for the index case of uncomplicated Graves' disease. USA and EU data are from references [5] and [6], respectively. ATD, antithyroid drug; EU, Europe; RAI, radioactive iodine; USA, United States of America.

A beta-blocker would initially be used definitely or possibly by the vast majority of respondents (90.8% and 7.5%, respectively). Propranolol was the preferred drug in 65% of the respondents, followed by atenolol in 32.5%. The target heart rate was 90–100 beats per minute for 40% of the respondents, 80–90 beats per minute for 34.2%, and 70–80 beats per minute for 23.3% of the respondents. ATD therapy was the preferred first-line approach (90.8%), and RAI treatment was selected as the initial treatment by only 9.2%, and thyroidectomy was not selected by any respondent (Figure 2). According to the practice settings and graduation years, there is no difference in the preferred therapy.

#### 3.3.2. ATD Treatment

Methimazole (MMI) was the preferred ATD for 100% of the respondents. It should be noted that carbimazole is not available in Thailand. The preferred starting dose of MMI was 10–15 mg once daily by 89.2% of the respondents, followed by 20 mg once daily (6.7%) and 30 mg once daily (3.3%). The most frequent starting doses of propylthiouracil (PTU) were 50 mg three times daily by 30% of the respondents, 100 mg three times daily (27.5%), and 150 mg three times daily (19.2%). The titration regimen was selected by 80.8% of the respondents, whereas the block-and-replace regimen was always used by 0.8% of respondents and in selected cases by 18.3%.

After initiating ATD therapy, the next measurement of serum thyroid hormone levels was performed after 4 weeks by 50.8% of respondents and after 6 weeks by 19.2%; after attaining euthyroidism, thyroid function tests would be most frequently performed every 2 (38.3%) or 3 (53.5%) months. Routine monitoring of CBCs and liver function tests during ATD treatment was performed by 19.1% and 5.8% of the respondents, respectively, whereas 80.9% of the respondents did not perform routine monitoring of either of these laboratory parameters.

In the case of a pruritic macular rash not responding to antihistamine therapy, 77.5% of the respondents switched to an alternate ATD, 12.5% continued with the same ATD with additional antihistamine therapy, and 10% selected an alternative treatment option for GD, including RAI or thyroid surgery. ATD therapy was continued for 18 months by the majority of respondents (45%), 27.5% continued ATD therapy for 24 months, and 12.5% continued ATD therapy for 12 months.

#### 3.3.3. Adjunctive ATD Treatment in Patients Receiving RAI

In patients receiving RAI therapy, premedication with ATDs was used routinely by 66.7% of the respondents, selectively used (commonly in patients >65 years old, with underlying heart disease or with multiple comorbidities) by 30.8%, and not used by 2.5%. When using premedication with ATDs before RAI, 64.2% withdrew ATDs at 7 days before RAI treatment, and 30.8% withdrew ATDs at 3–5 days before RAI treatment. In the early posttreatment phase, ATDs were routinely used by 72.5% of the respondents, used only selectively by 26.7%, and never used by 0.8%.

#### 3.3.4. Perioperative Management of Patients Undergoing Thyroidectomy

When thyroid surgery was selected, 95% of the respondents rendered patients in a state of biochemical euthyroidism with ATDs prior to surgery, whereas 5% would not. Preoperative iodine drops, either Lugol's solution or saturated solution of potassium iodide (SSKI), were used by 40% of the respondents. After surgery, prophylactic doses of calcium and/or vitamin D therapy at the time of discharge were not used by 69.2% if the postoperative calcium level was normal.

### 3.4. Variant 1: Hyperthyroidism with Concurrent GO or Risk Factors for GO

The index case was revised to include current cigarette smoking and the presence of moderately severe and active GO (Clinical Activity Score: 3 of 7 points; pain with eye movement, eyelid swelling, moderate conjunctival injection, and proptosis of 23 mm bilaterally). In this case, the majority of respondents (97.5%) received an ophthalmological consultation, and imaging evaluation of the orbit was requested by about 30% of the respondents (noncontrast computed tomography, 17.5%; magnetic resonance imaging, 12.5%; and ultrasound, 0.8%).

**Figure 3:**
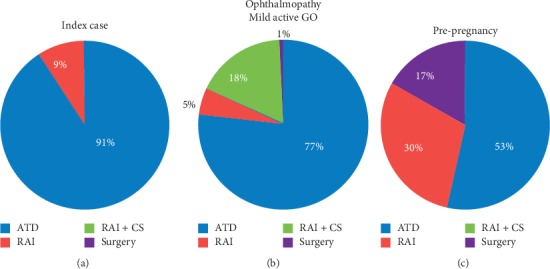
The effects of clinical variations on the selection of therapies for Graves' disease. (a) Uncomplicated Graves' disease (index case); (b) GO; (c) woman who planned to become pregnant in the next 6–12 months. ATD, antithyroid drug; CS, prophylactic corticosteroid therapy; GO, Graves' orbitopathy; RAI, radioactive iodine.

The preferred primary treatment method for hyperthyroidism in the presence of moderately severe and active GO was ATD treatment (62.5%). Thyroidectomy (after attaining euthyroidism with ATDs) was selected by 14.2% of the respondents. RAI treatment without steroids was not used by respondents, whereas 10.2% selected RAI plus low-dose glucocorticoids, and 12.5% used RAI with high-dose glucocorticoids (Figure 3 and Table 1).

In the presence of mild and active GO, ATDs were selected by 76.7% of the respondents, RAI alone by 5% of the respondents, RAI with low-dose glucocorticoids by 15%, and RAI with high-dose glucocorticoids by 2.5% of the respondents. If the patient had no signs of GO, but risk factors for the development of GO (smoking, high TRAb titers, and high serum T3 levels), responses did not change dramatically, except for the fact that no respondent would administer high-dose glucocorticoids if RAI treatment was the selected modality of treatment for hyperthyroidism (Table 1). Interestingly, in patients with sight-threatening GO, a slight majority of respondents (43.3%) recommended thyroid surgery after attaining euthyroidism with ATDs.

In the great majority of cases (70.8%), high-dose glucocorticoid treatment for active GO was administered by an ophthalmologist, and 26.7% was administered by an endocrinologist.

### 3.5. Variant 2: Hyperthyroidism Management in a Patient Planning a Pregnancy

The index case was then changed to a young woman planning a pregnancy over the next 6–12 months. ATDs were the preferred treatment option by 53% of the respondents, followed by RAI with 30% and thyroidectomy with 17% (Figure 3). In this situation, PTU was preferred by 63% of the respondents, and the remaining 37% preferred MMI. In addition, if the patient had a positive pregnancy test while on MMI treatment, the vast majority of the respondents (97.5%) shifted to PTU, but during the second and third trimesters, 67.5% of respondents switched back to MMI.

## 4. Discussion

The current study represents the perspectives of Thai endocrinologists in the management of GD. To the best of our knowledge, this is the first survey conducted in Southeast Asia. Previous data on Asia were mostly obtained from Japan [9]. However, the nations in the Asian continent have high heterogeneity in geography, ethnicity, and economic profile. This highlights the importance of country-specific information.

Measurement of TRAbs is a reliable and cost-effective laboratory investigation in the diagnosis of GD hyperthyroidism. Thyroid RAI uptake still offers definitive diagnostic imaging to determining the underlying cause of thyrotoxicosis. If a thyroid nodule is present, a thyroid scan should be added to determine the functional status of the nodule. Compared with North Americans and Europeans, the use of diagnostic tests for GD, such as TRAbs, isotopic studies were ordered less frequently in Thailand. TRAb measurement was used as diagnostic tool by 94.5% of the Korean respondents, 93.9% of the Italian respondents, 85.6% of the European respondents, 54.3% of the North American respondents, and only 29.2% of the Thai respondents [5, 6, 10, 11]. Moreover, thyroid ultrasound and isotopic studies were requested only by a small proportion of respondents in Thailand. Practicing medicine in resource-limited settings, such as Thailand, is challenging. Where laboratory access is limited and there are cost constraints in healthcare systems, most physicians use the clinical recognition of findings to direct decision making. Universal healthcare coverage has improved access to care, but inequality exists between different health plans [12].

The treatment selection for hyperthyroidism should take into account the balance of risk of harm and potential benefits for each available treatment option, in addition to patient preferences, health status, and access to treatment options. In our study, ATD therapy was the preferred treatment option (90.8% of respondents) for a first episode of hyperthyroidism. Accordingly, ATD therapy as the preferred treatment option for respondents from Korea (97%), Japan (88%), Europe (77%), Australia (81%), United Kingdom (60%), New Zealand (59%), and the Middle East and North Africa (53%) varied [5, 6, 11, 13–16]. RAI has been the preferred first-line treatment of North American clinicians. However, in recent decades, preference for RAI treatment has declined in favor of ATDs [5]. Fear of radiation is a main reason for the low preference of RAI treatment in Asia [7]. In addition, the increased risk of GO development or deterioration, as well as increasing concerns about the risks of radiation-induced cancer, was observed after RAI therapy. The risk of RAI-induced GO can be prevented by administration of oral or intravenous glucocorticoid [3, 17]. The recent data from a large, longitudinal cohort study showed RAI for hyperthyroidism could affect, in the long-term, increased I-131 dose-dependent mortality from solid cancers [18]. However, there were widespread criticisms on the previously mentioned study because of the lack of appropriate controls and novel nonvalidated analysis [19–22]. Several studies reported no correlation between the development of cancer and RAI [23–26]. Based on the current evidence, RAI treatment for GD is considered a safe procedure as recommended by ATA and ETA guidelines [2, 3]. Thyroidectomy is never selected in Thailand for the initial treatment of uncomplicated GD. Preference for initial thyroid surgery has remained low in many regions. Selection of surgery could be related to the fact that inevitable postoperative hypothyroidism requires less monitoring, regarding both follow-up visits and laboratory tests, than that during ATD therapy [27–29]. Moreover, thyroidectomy would be selected because of insufficiency of endocrinology and nuclear medicine centers in remote areas.

As most Thai endocrinologists followed the ATA guidelines, MMI was the only ATD recommended by the endocrinologists. After the 2011 guidelines were approved, MMI should have been used in virtually every patient, except during the treatment of a thyroid storm, in the first trimester of pregnancy, and in patients with minor allergic reactions to MMI [2]. This change in clinical practice results from the fact that PTU can induce fulminant hepatic necrosis, which might be lethal or require hepatic transplantation [30]. The results of this study were similar to other surveys [5, 6, 10, 11]. The preferred initial daily dose of MMI (15 mg/day) was lower than that reported in Caucasians [5, 6, 10]. A 15 mg dose of MMI not only resulted in a comparable inhibitory effect on thyroid function as those treated with a high dose (30 mg) of MMI in patients with GD but also caused fewer adverse effects [31]. However, the dose of MMI should be adjusted to disease severity because a dose that is too small is insufficient to restore euthyroidism in patients with severe hyperthyroidism [32]. Most respondents did not receive CBC tests during ATD therapy, corresponding with the ATA and ETA recommendations [2, 3]. In Japan, routine monitoring of CBCs is recommended during the first 2 months of ATD therapy [32].

From the ATA and ETA guidelines, preoperative Lugol's solution or SSKI should be given prior to thyroidectomy in most patients with GD. This treatment is useful because it reduces thyroid vascularity and intraoperative bleeding during thyroid surgery [33]. However, this protocol is used only by approximately one-third of endocrinologists [5, 6, 10]. Approximately 30% of the respondents considered prophylactic treatment with oral calcium with or without oral calcitriol. As mentioned in the ATA statement, this approach is cost-effective and can hasten hospital discharge [34, 35].

GO is the main extrathyroidal manifestation of GD, although fortunately, severe forms are rare. When GD is complicated by moderately severe and active orbitopathy, the majority of Thai endocrinologists first consult with an ophthalmologist. This is similar to colleagues in other countries [5, 6, 10]. However, steroids were administered by Thai endocrinologists to only 26.7% of the patients. This study revealed that the majority of respondents would treat patients who have associated GO with ATDs. There was a more than 10-fold increased use of thyroidectomy when the index case was modified for a patient with moderate GO. Patients with moderate-to-severe active GO should receive prompt treatment using high-dose systemic glucocorticoids [36, 37]. Almost one-third of the respondents proceeded to the ablative approach by either RAI or thyroidectomy. In a patient with mild active GO, most respondents related the opportunity for concurrent steroid prophylaxis with low-dose oral prednisone and indicated if RAI treatment was selected, as recommended by the European Group on GO [37].

If a GD woman under ATD treatment wished to become pregnant in the next 6–12 months, most respondents treated with an ATD, with a preference for PTU over MMI. This approach may minimize prenatal MMI exposure during the sensitive period of organogenesis. Conversely, definitive treatment by surgery was the treatment of choice for a women planning pregnancy by half of the Italian respondents [10]. The advantage of thyroidectomy is the gradual remission of circulating TRAbs occurring postsurgery [38]. Despite the fact that RAI will transiently raise TRAb titers for months to years, which may contribute to worsening GO or fetal risk [39, 40], RAI was the second choice of treatment in the present study and the North American survey [5]. There was a similar pattern between other regions in the preference for PTU during the first trimester of pregnancy, as well as in the decision to replace the treatment with MMI in the second and third trimesters. The majority of our respondents followed this approach, which is recommended by ATA guidelines [38].

In conclusion, geographical differences exist in the diagnosis and management of GD. These differences in treatment options may be caused by the availability of nuclear medicine facilities and experienced thyroid surgeons. According to the substantial practice variations in the diagnosis and management of GD in Thailand, compared with those in other countries, additional detailed studies investigating the cost- and risk-effective management of GD are needed.

## Figures and Tables

**Table 1:** Percentage of respondents choosing the preferred treatment modalities for the index case when GO occurs.

	No signs of GO; only risk factors (%)	Mild active GO (%)	Moderately severe and active GO (%)
ATD	78.3	76.7	62.5
Thyroidectomy	0.8	0.8	14.2
RAI alone	15	5	0
RAI with low-dose steroid	5	15	10.2
RAI with high-dose steroid	0.8	2.5	12.5

Abbreviation: ATD, antithyroid drug; GO, Graves' orbitopathy; RAI, radioactive iodine.

## Data Availability

The datasets generated during and/or analysed during the current study are available from the corresponding author upon reasonable request.
